# P-681. Estimated Incidence of Respiratory Syncytial Virus (RSV)-related Hospitalizations for Acute Respiratory Infections (ARIs), including Community Acquired Pneumonia (CAP), in Adults in Germany

**DOI:** 10.1093/ofid/ofae631.877

**Published:** 2025-01-29

**Authors:** Caihua Liang, Elizabeth Begier, Liz Wang, Claudia Schwarz, Lea Johanna Bayer, Christof Von Eiff, Qing Liu, Jo Southern, Jeffrey T Vietri, Sonal Uppal, Bradford D Gessner, Christian Theilacker

**Affiliations:** Pfizer Inc, New York, New York; Pfizer Vaccines, Dublin, Dublin, Ireland; Pfizer, New York, New York; RWE Platform, Vienna, Wien, Austria; Pfizer Pharma GmbH, Berlin, Berlin, Germany; Pfizer Pharma gmbH, Berlin, Berlin, Germany; Pfizer Inc., Collegeville, Pennsylvania; Pfizer Inc, New York, New York; Pfizer, Inc., Collegeville, Pennsylvania; Pfizer, New York, New York; Pfizer Biopharma Group, Collegeville, Pennsylvania; Pfizer Inc., Collegeville, Pennsylvania

## Abstract

**Background:**

RSV is a leading cause of ARI, including CAP, in older adults, but available data are limited and often substantially underestimate incidence. Among adults, single nasopharyngeal (NP) swab testing misses about half of RSV infections compared to using multiple specimen types. We estimated RSV-related ARI hospitalization incidence from a prospective CAP study and adjusted for undiagnosed RSV infections due to use of NP swab testing only.

**Figure:**
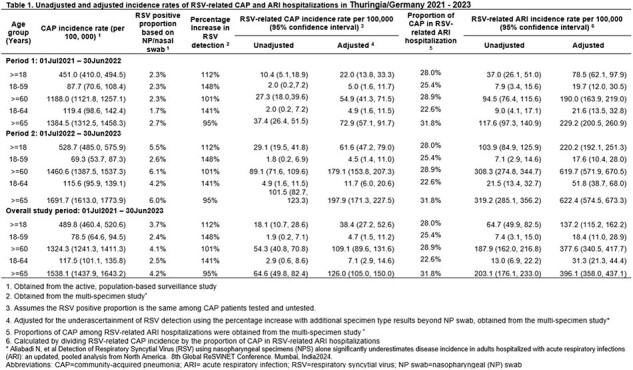

**Methods:**

We conducted an active, population-based multicenter surveillance of adult CAP hospitalizations in Thuringia (Germany) between 2021–2023. Participant NP swabs were RSV-tested by multiplex nucleic acid amplification tests. To estimate unadjusted RSV-related CAP incidence, age-group specific proportions of RSV detection among tested patients were applied to all-cause CAP incidence. Adjustments for underdiagnosis due to single site sampling and exclusion of non-CAP ARI were based on previous findings from a large, prospective, multispecimen study assessing the impact of collecting multiple specimens (NP swab, saliva, paired serology, and sputum) among 3,669 adults hospitalized for ARI.

**Results:**

Among 1,040 radiologically confirmed CAP adults enrolled, 38 tested positive for RSV based on NP swabs, for an overall positive proportion of 3.7% among adults aged ≥18 years. Positive proportion increased to 7.8% after adjusting for higher RSV detection with multispecimen sampling compared to NP swab sampling only. The adjusted annual RSV-related CAP hospitalization rates were 4.7 (95% CI 1.5, 11.2) and 109.1 (95% CI 89.6, 131.6) per 100,000 population in adults aged 18-59 and ≥60 years, respectively. As CAP comprises only 28% of RSV-related ARI, the adjusted annual incidence of RSV-related ARI was 18.4 (95% CI 11.0, 28.9) and 377.6 (95% CI 340.5, 417.7) per 100,000 population for adults 18-59 and ≥60 years, respectively (Table1). Incidence estimates in Period 2 were about 3 times higher than in Period 1.

**Conclusion:**

Older adults in Germany face a high burden of RSV-related ARI hospitalizations, including CAP, underscoring the potential utility of RSV vaccination for this population. These results align with recent time-series incidence analysis from Germany (236–363/100,000 for adults ≥60 years).

**Disclosures:**

**Caihua Liang, MD, PhD**, Pfizer: Stocks/Bonds (Private Company) **Elizabeth Begier, MD, M.P.H.**, Pfizer Vaccines: Employee|Pfizer Vaccines: Stocks/Bonds (Private Company) **Liz Wang, MD; MS Biostatistics**, Pfizer: employee|Pfizer: Stocks/Bonds (Public Company) **Claudia Schwarz, PhD**, Pfizer Inc.: employee|Pfizer Inc.: Stocks/Bonds (Private Company) **Lea Johanna Bayer, n/a**, Pfizer Pharma: Employee **Christof Von Eiff, n/a**, Pfizer Pharma: I am working as Sen. Medical Direktor for Vaccines at Pfizer Pharma GmbH, Berlin, , Germany and in that position I also get stocks. **Qing Liu, M.S.**, Pfizer Inc.: Stocks/Bonds (Public Company) **Jo Southern, PhD, MSc**, Pfizer: Employee|Pfizer: Stocks/Bonds (Private Company) **Jeffrey T. Vietri, PhD**, Pfizer Inc.: Employment|Pfizer Inc.: Stocks/Bonds (Public Company) **Bradford D. Gessner, M.D., M.P.H.**, Pfizer: Employee|Pfizer: Stocks/Bonds (Public Company) **Christian Theilacker, MD, DTM&H**, Pfizer Inc: I am a Pfizer Employee|Pfizer Inc: Stocks/Bonds (Public Company)

